# Living alone is associated with a higher prevalence of psychiatric morbidity in a population-based cross-sectional study

**DOI:** 10.3389/fpubh.2022.1054615

**Published:** 2022-11-17

**Authors:** Te-Yu Chen, Jiun-Hung Geng, Szu-Chia Chen, Jia-In Lee

**Affiliations:** ^1^School of Post-Baccalaureate Medicine, College of Medicine, Kaohsiung Medical University, Kaohsiung, Taiwan; ^2^Department of Urology, Kaohsiung Municipal Siaogang Hospital, Kaohsiung, Taiwan; ^3^Department of Urology, Kaohsiung Medical University Hospital, Kaohsiung Medical University, Kaohsiung, Taiwan; ^4^Graduate Institute of Clinical Medicine, College of Medicine, Kaohsiung Medical University, Kaohsiung, Taiwan; ^5^Research Center for Environmental Medicine, Kaohsiung Medical University, Kaohsiung, Taiwan; ^6^Department of Internal Medicine, Kaohsiung Municipal Siaogang Hospital, Kaohsiung Medical University, Kaohsiung, Taiwan; ^7^Division of Nephrology, Department of Internal Medicine, Kaohsiung Medical University Hospital, Kaohsiung Medical University, Kaohsiung, Taiwan; ^8^Faculty of Medicine, College of Medicine, Kaohsiung Medical University, Kaohsiung, Taiwan; ^9^Graduate Institute of Medicine, College of Medicine, Kaohsiung Medical University, Kaohsiung, Taiwan; ^10^Department of Psychiatry, Kaohsiung Medical University Hospital, Kaohsiung Medical University, Kaohsiung, Taiwan

**Keywords:** dependence, depression, anxiety, Psychiatric disorders, psychiatric distress, psychiatric morbidities, living alone

## Abstract

**Background:**

Living alone has been linked to poor mental health, however large-scale epidemiological studies on the association between living alone and psychiatric morbidity including depression and anxiety are lacking. The aim of this study was to investigate this issue in a large Taiwanese cohort.

**Methods:**

In this cross-sectional study, we enrolled 121,601 volunteers from 29 community recruitment stations in Taiwan and divided them into two groups based on whether or not they lived alone. Psychiatric morbidity was defined as a Generalized Anxiety Disorder 2-item score ≥ 3, Patient Health Questionnaire 2-item score ≥ 3, or self-reported depression. Logistic regression was used to explore the associations between living alone and psychiatric morbidity.

**Results:**

The participants who lived alone had a higher prevalence of psychiatric morbidity [odds ratio (OR) = 1.608, 95% confidence interval (CI) = 1.473 to 1.755] after adjusting for potential confounders. In a subgroup analysis, married subjects who lived alone and divorce/separation (OR = 2.013, 95% CI = 1.763 to 2.299) or widowing (OR = 1.750, 95% CI = 1.373 to 2.229) were more likely to have psychiatric morbidity than those who were married and not living alone.

**Conclusions:**

Our findings suggest that living alone is a risk factor for psychiatric morbidity, especially for married subjects who live alone in concordance with divorce, separation, or the death of a spouse.

## Introduction

In recent years, the increase in unmarried, later marriage, and divorce rates has caused the pattern of marriage and family to change dramatically. A smaller family size has led to a growth in single-person households (1), and social isolation and a lack of contact has been associated with health issues such as mental disorders, dementia, poor nutrition, and cardiac disease (2–4). Worldwide, more than 300 million people are affected by depression, and more than 250 million live with anxiety disorders (5). According to a cross-sectional survey in Taiwan, the prevalence of potentially common mental diseases, including non-psychotic, depression, and anxiety disorders, doubled from 11.5% in 1990 to 23.8% in 2010 (6). Proper care and treatment for these patients can reduce mortality and extend life expectancy (7). Moreover, an increase in the prevalence of these common mental diseases would result in tremendous medical and social costs (8, 9), making it crucial to determine the risk factors and comorbidities associated with them.

Researchers have investigated the relationship between living alone and mental health, and found that social isolation increases the risk of common mental diseases (10). However, these studies mainly focused on the effects of depression on elderly populations, rather than on the general or young populations. In addition, only a few have mentioned the association between living alone and other psychiatric conditions, such as anxiety, and most only included a small number of subjects (11). Furthermore, as the number of single-person households increases, mental disorders could also affect younger people who are unmarried or divorced as well as elderly populations.

Psychiatric morbidity is a symptom-based medical term generally applied to those aware of their condition, including a variety of mental illnesses, such as depression, anxiety, schizophrenia, bipolar disorder, et cetera, which is well–suited for exploring the association between living alone and mental health (12). One advantage of using psychiatric morbidity to test our hypothesis is that it not only covers depression, but also other psychiatric disorders as well. Another advantage is that psychiatric morbidity often represents the symptomatic group, which makes our results more clinically meaningful (12). Because living alone has been associated with loneliness (13), social support (14), and substance use (15), which are risk factors for psychiatric morbidity, we hypothesize that living alone is associated with psychiatric morbidity. Previously, numerous studies have documented that education (16), smoking (17), drinking (18), chronic disorders (19), stressful life events (20) and obesity (21) are associated with psychiatric morbidity; however, only a few have mentioned the association between living alone and psychiatric morbidity, and most only included a small number of subjects (10). The goals of this study were to determine the association between living alone and psychiatric morbidity.

## Materials and methods

### Data source and study population

The data used in this study were from a population-based dataset derived from 29 community recruitment stations in Taiwan since 2008, details of which have been described in our previous publications (22–24). In brief, all subjects were enrolled as volunteers and completed several questionnaires including basic profile, habitus, past history, Generalized Anxiety Disorder 2-item (GAD-2) and Patient Health Questionnaire 2-item (PHQ-2). They also underwent physical examinations during which body weight and height were measured and blood tests were performed. Before enrollment, all subjects understood the purpose, interests, pros and cons of our research and signed a consent form. All researchers followed the Declaration of Helsinki throughout the study, which was approved by the Institutional Review Board of our institute (KMUHIRB-E(I)-20210058).

### Variables

The variables used in this study came from the aforementioned questionnaires, physical examinations, and blood tests. Data on age, gender, smoking status, drinking status, exercise status, marital status, educational status, medical history, GAD-2 and PHQ-2 scores, were obtained from the questionnaires. Data on body mass index (BMI) and waist circumference were obtained from the physical examinations, and data on serum creatinine and chronic kidney disease (defined as an estimated glomerular filtration rate < 60 ml/min/1.73 m^2^) were obtained from the blood tests.

### Living arrangements and marital status

Each subject would need to fill out a questionnaire about their living arrangements and marital status. Regarding living arrangements, there are two options in the questionnaire: (1) living alone and (2) living with family or others. Based on the subjects' responses, they were divided into living alone (+) and living alone (-). Regarding marital status, there are four options in the questionnaire: (1) unmarried (single/never married), (2) married, (3) divorced or separated, and (4) widowed. We further combined living arrangements with marital status, and subjects were subdivided into 6 groups: (1) living alone (+) and unmarried, (2) living alone (+) and married, (3) living alone (+) and divorce/separation, (4) living alone (+) and widowing, (5) living alone (-) and unmarried, (6) living alone (-) and married.

### Psychiatric morbidity

Psychiatric morbidity was defined as depression and anxiety in this study. We used the PHQ-2, GAD-2, and self-reported depression to assess the presence or absence of psychiatric morbidity. The PHQ-2 uses the following two questions to assess a subject's depressive condition in the last 2 weeks: “Do you feel little interest or pleasure in doing things? (0 = not at all; 1 = several days; 2 = more than half the days; 3 = nearly every day)” and “Do you feel down, depressed, or hopeless? (0 = not at all; 1 = several days; 2 = more than half the days; 3 = nearly every day)”. The GAD-2 also uses two questions to assess a subject's anxiety in the last 2 weeks as follows: "Do you feel nervous, anxious, or on edge? (0 = not at all; 1 = several days; 2 = more than half the days; 3 = nearly every day)” and “Do you feel unable to stop or control worrying? (0 = not at all; 1 = several days; 2 = more than half the days; 3 = nearly every day)”. Participants with a PHQ-2 score of 3 to 6 were considered to have depressive tendencies, and those with a GAD-2 score of 3 to 6 were considered to have anxiety tendencies. We further defined psychiatric morbidity as a GAD-2 score ≥ 3, PHQ-2 score ≥ 3, or self-reported depression.

### Statistical analysis

We used descriptive statistics to describe the profiles of all subjects. Continuous variables are expressed as means and standard deviations, and categorical variables are expressed as numbers and percentages. We then divided the subjects into two groups according to whether or not they lived alone, and the differences between groups were measured using the independent *t* test and chi-square test. The possible confounders of the association between living alone and psychiatric morbidity were identified through literature reviews, including age (25), sex (25), obesity (21), educational status (16), smoking (17), drinking (18), physical activity (26), marital status (27) and chronic diseases (heart disease, asthma, chronic obstructive pulmonary disease, gastrointestinal problems, hypertension, diabetes mellitus, dyslipidemia, gout, osteoporosis, chronic kidney disease, neurological diseases and substance abuse) (19, 28–30). To further identify the risk factors, these possible confounders were entered into the feature selection process using the least absolute shrinkage and selection operator (LASSO) regression, by assigning each training observation to be subdivided randomly into 10 parts, then by a method of an automated 10-fold cross-validation (31). We further used univariable and multivariable logistic regression to test the association of each variable with psychiatric morbidity. Finally, we conducted a subgroup analysis to explore the association of marriage and dependency with psychiatric morbidity. In this study, a *p* value < 0.05 indicated a significant association. Our analysis was performed using R (version 3.6.2, R Foundation for Statistical Computing, Wien, Austria), SAS (version 9.4, SAS Institute Inc., Cary, NC, United States) and SPSS (version 20.0, IBM Corp, Armonk, NY, United States).

## Results

### Profiles of the participants

A total of 121,601 participants with sufficient data were included in our analysis. Their average age was 50 years, 27.3% smoked, 8.5% drank alcohol, 86.4% were married, 3.6% had self-reported diagnosed depression, 4.5% had psychiatric morbidity and 8.1% lived alone (Table 1). Among the 16,582 participants who completed the PHQ-2 and GAD-2, the average PHQ-2 score was 0.53, the GAD-2 score was 0.56, 4.2% had a PHQ-2 ≧ 3, and 5.4% had a GAD-2 ≧ 3. The subjects' past medical histories are also listed in Table 1. The subjects who lived alone (*n* = 9,828) had a lower BMI, lower waist circumference, higher proportion of smoking, drinking, PHQ-2 ≧ 3, GAD-2 ≧ 3, psychiatric morbidity, and more were unmarried than those who did not live alone. Concerning past medical history, the subjects who lived alone were more likely to have diabetes, respiratory diseases, gastrointestinal diseases, orthopedic diseases, neurological diseases and self-reported diagnosed depression than those who did not live alone (Table 1).

**Table 1:** Profiles of participants.

**Characteristics**	**Total** **(*n* = 121,601)**	**Living alone (+)** **(*n* = 9,828)**	**Living alone (-)** **(*n* = 111,773)**	***p* value**
Age, yr	50 ± 11	50 ± 12	50 ± 11	0.311
Male gender, *n* (%)	43,699 (35.9)	3,000 (30.5)	40,699 (36.4)	<0.001
Body mass index, kg/m2	24.2 ± 3.8	24.0 ± 4.0	24.2 ± 3.8	<0.001
Waist circumference, cm	83.3 ± 10	82.7 ± 10.8	83.4 ± 10.2	<0.001
Smoking status, ever, *n* (%)	33,156 (27.3)	2,765 (28.1)	30,391 (27.2)	0.044
Drinking status, ever, *n* (%)	10,357 (8.5)	892 (9.1)	9,465 (8.5)	0.039
Regular exercise, yes, *n* (%)	49,304 (40.5)	4,073 (41.4)	45,231 (40.5)	0.059
Marital status, married, *n* (%)	105,059 (86.4)	5,409 (55.0)	99,650 (89.2)	<0.001
Education status, ≧College, *n* (%)	70,475 (58.0)	6,054 (61.6)	64,421(57.6)	<0.001
Hypertension, *n* (%)	14,887 (12.2)	1,185 (12.1)	13,702 (12.3)	0.574
Diabetes, *n* (%)	6,276 (5.2)	569 (5.8)	5,707 (5.1)	0.003
Dyslipidemia, *n* (%)	9,041 (7.4)	759 (7.7)	8,282 (7.4)	0.261
CAD, *n* (%)	1,562 (1.3)	142 (1.4)	1,420 (1.3)	0.146
Valvular heart disease, *n* (%)	5,092 (4.2)	513 (5.2)	4,579 (4.1)	<0.001
Asthma. *n* (%)	4,301 (3.5)	408 (4.2)	3,893 (3.5)	0.001
COPD, *n* (%)	1,390 (1.1)	154 (1.6)	1,236 (1.1)	<0.001
Osteoporosis, *n* (%)	4,994 (4.1)	535 (5.4)	4,459 (4.0)	<0.001
Gout, *n* (%)	4,675 (3.8)	289 (2.9)	4,386 (3.9)	<0.001
GERD, *n* (%)	16,666 (13.7)	1,463 (14.9)	15,203 (13.6)	<0.001
Peptic ulcer, *n* (%)	17,701 (14.6)	1,531 (15.6)	16,170 (14.5)	0.003
IBS, *n* (%)	3,026 (2.5)	275 (2.8)	2,751 (2.5)	0.041
CKD, *n* (%)	1,951 (1.6)	178 (1.8)	1,773 (1.6)	0.092
Parkinson's disease, *n* (%)	131 (0.1)	11 (0.1)	120 (0.1)	0.872
Dementia, *n* (%)	37 (0.2)	7 (0.1)	30 (0.0)	0.027
Schizophrenia, *n* (%)	237 (0.2)	34 (0.3)	203 (0.2)	0.001
Substance abuse, *n* (%)	44 (0.0)	9 (0.1)	35 (0.0)	0.008
Self-reported depression	4,362 (3.6)	607 (6.2)	3,755 (3.4)	<0.001
PHQ-2	0.53 ± 0.99	0.73 ± 1.15	0.51 ± 0.97	<0.001
PHQ-2 ≧ 3*	699 (4.2)	87 (6.4)	612 (4.0)	<0.001
GAD-2	0.56 ± 0.97	0.73 ± 1.15	0.54 ± 0.96	<0.001
GAD-2 ≧ 3*	896 (5.4)	117 (8.6)	779 (5.0)	<0.001
Psychiatric morbidity	5,414 (4.5)	729 (7.4)	4,685 (4.2)	<0.001

CAD, coronary artery disease; COPD, chronic obstructive pulmonary disease; GERD, gastroesophageal reflux disease; IBS, irritable bowel syndrome; CKD, chronic kidney disease; PHQ-2, Patient Health Questionnaire 2-item; GAD-2, Generalized Anxiety Disorder 2-item.

* There were 16,582 participants providing data on GAD-2 and PHQ-2.

### Parameters associated with psychiatric morbidity in univariable binary logistic analysis

By performing LASSO regression, we excluded three variables, including drinking status, exercise and chronic kidney disease, that had minimal effects on psychiatric morbidity (Supplementary Figure 1 and Supplementary Table 1). The remaining 24 variables were entered into further analysis. In univariable binary logistic analysis, female gender, smoking, diabetes mellitus, dyslipidemia, cardiovascular diseases, respiratory diseases, orthopedic diseases, gastrointestinal diseases, neurological diseases, and living alone were associated with a higher prevalence of psychiatric disorders (Table 2). The subjects who lived alone had a higher prevalence of psychiatric morbidity with an odds ratio (OR) of 1.831 [95% confidence interval (CI) = 1.689 to 1.986, *p* < 0.001]. Conversely, being married and having a high degree of education were associated with a lower prevalence of psychiatric disorders (Table 2).

**Table 2:** Parameters associated with psychiatric morbidity in univariable binary logistic analysis.

**Parameters**	**Odds ratio (95% CI)**	***P* value**
Age (per 1 year)	1.002 (1.000 to 1.005)	0.086
Female gender (vs. *male gender*)	1.683 (1.581 to 1.791)	< 0.001
Body mass index (per 1 kg/m^2^)	0.996 (0.989 to 1.003)	0.277
Waist circumference (per 1 cm)	1.001 (0.999 to 1.004)	0.338
Smoking status, ever (vs. never)	1.244 (1.173 to 1.319)	<0.001
Marital status, married (vs. no)	0.805 (0.747 to 0.867)	<0.001
Education status, ≧College (vs. no)	0.846 (0.809 to 0.884)	<0.001
Hypertension, yes (vs. no)	1.264 (1.171 to 1.366)	<0.001
Diabetes mellitus, yes (vs. no)	1.485 (1.336 to 1.650)	<0.001
Dyslipidemia, yes (vs. no)	1.700 (1.561 to 1.853)	<0.001
CAD, yes (vs. no)	1.804 (1.494 to 2.177)	<0.001
Valvular heart disease, yes (vs. no)	2.115 (1.910 to 2.342)	<0.001
Asthma, yes (vs. no)	2.016 (1.802 to 2.254)	<0.001
COPD, yes (vs. no)	2.128 (1.766 to 2.564)	<0.001
Osteoporosis, yes (vs. no)	1.853 (1.664 to 2.064)	<0.001
Gout, yes (vs. no)	0.768 (0.656 to 0.900)	0.001
GERD, yes (vs. no)	2.244 (2.106 to 2.391)	<0.001
Peptic ulcer, yes (vs. no)	2.048 (1.921 to 2.182)	<0.001
IBS, yes (vs. no)	3.023 (2.698 to 3.387)	<0.001
Parkinson's Disease, yes (vs. no)	3.877 (2.407 to 6.247)	<0.001
Dementia, yes (vs. no)	9.096 (4.492 to 18.418)	<0.001
Schizophrenia, yes (vs. no)	6.857 (5.085 to 9.249)	<0.001
Substance abuse, yes (vs. no)	7.166 (3.620 to 14.186)	<0.001
**Living alone, yes (vs. no)**	1.831 (1.689 to 1.986)	<0.001

Abbreviations are as Table 1, CI, Confidence interval.

### Parameters associated with psychiatric morbidity in multivariate binary logistic analysis

In multivariable binary logistic analysis, female gender, waist circumference, smoking, diabetes mellitus, dyslipidemia, cardiovascular diseases, respiratory diseases, orthopedic diseases, gastrointestinal diseases, neurological diseases, and living alone were associated with a higher prevalence of psychiatric disorders (Table 3). The subjects who lived alone had a higher prevalence of psychiatric morbidity with an OR of 1.608 (95% CI = 1.473 to 1.755, *p* < 0.001) after adjusting for potential confounders. In contrast, age, BMI, being married, and having a high degree of education were associated with a lower prevalence of psychiatric disorders (Table 3). We further analyzed males and females separately, and the results were similar to the results of the whole study population (Supplementary Table 2).

**Table 3:** Parameters associated with psychiatric morbidity in multivariate binary logistic analysis.

**Parameters**	**Odds ratio (95% CI)**	***p* value**
Age (per 1 year)	0.992 (0.989 to 0.995)	<0.001
Female gender (vs. *male gender*)	2.359 (2.180 to 2.553)	<0.001
Body mass index (per 1 kg/m^2^)	0.966 (0.953 to 0.979)	<0.001
Waist circumference (per 1 cm)	1.016 (1.010 to 1.021)	<0.001
Smoking status, ever (vs. never)	1.771 (1.650 to 1.900)	<0.001
Marital status, married (vs. no)	0.899 (0.824 to 0.980)	0.015
Education status, ≧College (vs. no)	0.856 (0.806 to 0.909)	<0.001
Hypertension, yes (vs. no)	1.184 (1.084 to 1.292)	<0.001
Diabetes mellitus, yes (vs. no)	1.263 (1.125 to 1.417)	<0.001
Dyslipidemia, yes (vs. no)	1.358 (1.234 to 1.495)	<0.001
CAD, yes (vs. no)	1.397 (1.143 to 1.708)	0.001
Valvular heart disease, yes (vs. no)	1.569 (1.411 to 1.744)	<0.001
Asthma, yes (vs. no)	1.632 (1.453 to 1.834)	<0.001
COPD, yes (vs. no)	1.482 (1.218 to 1.803)	<0.001
Osteoporosis, yes (vs. no)	1.418 (1.264 to 1.591)	<0.001
Gout, yes (vs. no)	0.844 (0.713 to 0.999)	0.048
GERD, yes (vs. no)	1.661 (1.550 to 1.780)	<0.001
Peptic ulcer, yes (vs. no)	1.618 (1.510 to 1.733)	<0.001
IBS, yes (vs. no)	2.375(2.109 to 2.674)	<0.001
Parkinson's Disease, yes (vs. no)	2.750 (1.620 to 4.669)	<0.001
Dementia, yes (vs. no)	5.679 (2.586 to 12.472)	<0.001
Schizophrenia, yes (vs. no)	5.435 (3.932 to 7.513)	<0.001
Substance abuse, yes (vs. no)	4.178 (1.960 to 8.908)	<0.001
**Living alone, yes (vs. no)**	1.608 (1.473 to 1.755)	<0.001

Abbreviations are as Table 1, CI, Confidence interval.

Covariates in the multivariable model included age, gender, body mass index, waist circumference, smoking status, married status, educational status, hypertension, diabetes mellitus, dyslipidemia, coronary artery disease, valvular heart disease, asthma, chronic obstructive pulmonary disease, osteoporosis, gout, gastroesophageal reflux disease, peptic ulcer, irritable bowel syndrome, Parkinson's Disease, dementia, schizophrenia and substance abuse.

### Odds ratios for psychiatric morbidity by marital status

Because the presence or absence of living alone is related to marital status, we then performed a subgroup analysis, and divided the subjects into two groups according to marital status. As shown in Supplementary Table 3, the risk of psychiatric morbidity in the unmarried group was not affected by living alone (OR = 1.155, 95% CI = 0.990 to 1.348, *p* = 0.067), however the risk of psychiatric disorders in the married group was related to living alone (OR = 1.876, 95% CI = 1.692 to 2.081, *p* < 0.001). The results were similar when analyzing men and women separately (Supplementary Table 4). Moreover, married subjects who lived alone and divorce/separation (OR = 2.013, 95% CI = 1.763 to 2.299, *p* < 0.001) or widowing (OR = 1.750, 95% CI = 1.373 to 2.229, *p* < 0.001) were more likely to have psychiatric morbidity than those who were married and not living alone (Table 4 and Supplementary Table 5).

**Table 4:** Odds ratios for psychiatric morbidity by marital status.

**Characteristics**	**No. of psychiatric morbidity cases/ no. of subjects (%)**	**Adjusted odds ratio (95% CI)**	***p value***
**All subjects, unmarried (*****n*** **=** **16,542)**			
Living alone (-)	619/12,123 (5.1)	reference	–
Living alone (+)	259/4,419 (5.9)	1.155 (0.990 to 1.348)	0.067
**All subjects, married (*****n*** **=** **105,059)**			
Living alone (-)	4,066/99,650 (4.1)	reference	–
Living alone (+) and married	74/1,088 (6.8)	1.688(1.368 to 2.056)	<0.001
Living alone (+) and widowing	120/1,392 (8.6)	1.750 (1.373 to 2.229)	<0.001
Living alone (+) and divorce or separation	276/2,929 (9.4)	2.013 (1.763 to 2.299)	<0.001

CI, Confidence interval.

Covariates in the multivariable model included age, gender, body mass index, waist circumference, smoking status, educational status, hypertension, diabetes mellitus, dyslipidemia, coronary artery disease, valvular heart disease, asthma, chronic obstructive pulmonary disease, osteoporosis, gout, gastroesophageal reflux disease, peptic ulcer, irritable bowel syndrome, Parkinson's Disease, dementia, schizophrenia and substance abuse.

## Discussion

This study is the largest population-based study to examine the association between living alone and psychiatric morbidity, and it showed a statistically significant association between them. We also found that this association was present in the subjects who were married, but not in those who were not married. In addition, married subjects who lived alone in concordance with divorce, separation, or the death of a spouse were associated with a higher risk of psychiatric morbidity.

Living alone has been associated with poor mental health conditions (32–36). Stahl, et al. (32) found that living alone was associated with elevated levels of depressive symptoms compared to living with a family member. Similar findings were also noted in the elderly population (34). In a qualitative meta-analysis, Hu, et al. found that older people living alone had a higher risk of depression than those not living alone (33). Consistent with our results, the relationship between living alone and anxiety has also been discussed in previous research; for example, Hunt, et al. (35) and Yu, et al. (36) found that people living alone had a significantly higher risk of generalized anxiety disorder than those living with their families.

An interesting finding of this study is that living alone increased the risk of psychiatric morbidity in married subjects, but not in unmarried subjects. A previous study reported that the psychological well-being of divorced and widowed people was poorer than those who never married (37). Marital relationships can provide a sense of well-being and emotional support, producing mutual obligations and reinforcements between the two parties (38, 39). These relationships reduce vulnerability to psychological disorders. However, a change in this connection has been shown to significantly increase depressive symptoms (40). Thus, this might explain a higher likelihood of married people but living alone suffering from psychiatric disorders.

We also found that married subjects who lived alone and widowed had a 1.76-fold risk of having psychiatric morbidity compared to those who were married and not living alone. Widowhood has been known for being a catastrophic event with a negative impact on both physical and emotional well-being (41, 42). Various factors have influenced the degree of emotional response to spouse loss such as age, gender, length of widowhood, health condition, economic status, and living arrangements (43, 44). Srivastava, et al. (45) reported that the interaction between marital status and living arrangements on depression showed that widowed and living alone elderly were more likely to suffer from depression than those currently married and co-residing. The rates of depression were highest in widowed and living alone, followed by widowed and co-residing, currently married and co-residing, currently married and living alone (45). The negative psychological well–being of widowhood could be explained by the poor emotional and financial support that comes with spouse loss (46, 47).

Meanwhile, the relationship between living alone and lack of social support was also reported in COVID-19 related studies that resulted in higher risk of depression and anxiety (48, 49). The addition of widowed and separated status was revealed to be related to depression and poor quality of life due to loneliness (50). Poor psychological well-being has been linked to objective social isolation (51) and subjectively perceived social support, such as loneliness (52). Moreover, in a large nationally longitudinal study, Domènech-Abella, et al. (53) reported that both loneliness and social isolation affected the probability of suffering from depression and anxiety.

This is an important public health issue, because people suffered from psychiatric disorders are at increased risk of suicide, self-harm, and mortality (54–56). Thus, proper care and treatment are crucial to reduce mortality and extend life expectancy. Stahl, et al. (32) suggested that adults living alone need to have a better perception of neighborhood social quality. Having a good relationship with neighbors has been shown to relieve loneliness and depression by increasing the availability of social activities, receiving practical help from others, and making older people feel safer and more secure (57–59). Another study showed that leisure activities may moderate poor mental health in older adults living alone (60). Older adults living alone may have reduced physical activity and social interaction, and encouraging them to participate in leisure activities could increase their level of physical activity and social connection with others and affect positive emotional outcomes (60–62). Thus, people living alone tend to have fewer social interactions and activities and feel lonely and insecure (36). Having a good relationship with neighbors or participating in leisure activities could reduce the risk of suicide, self-harm, and mortality.

Besides living alone, other parameters are also associated with psychiatric morbidity. Our results revealed that the subjects with chronic diseases, such as diabetes mellitus, cardiovascular, respiratory, orthopedic, gastrointestinal, or neurological diseases had a higher likelihood of psychiatric morbidity (63–66). Chaudhry, et al. (67) reported a prevalence of psychiatric morbidity among insulin-dependent patients of 18%, and that people with diabetes mellitus were twice as likely as the general population to suffer from psychiatric morbidity. Psychiatric morbidity is common in patients with coronary heart disease, and a previous study reported that 16% of patients assessed seven days after myocardial infarction had symptoms consistent with a major depressive episode (68, 69). With regards to the relationship between respiratory diseases and psychiatric morbidity, a study in India found that 44.8% of patients with respiratory illnesses had a mental illness compared with 24.3% of controls (70).

The relationship between psychiatric morbidity and chronic diseases could be attributed to patients' panic, pessimism, and emotional imbalance after diagnosis. From the aspect of biology, the hypothalamic adrenocortical axis could be induced by both depression and diabetes (67). Devolving psychiatric morbidity, hypertension, and cardiovascular diseases could be attributed to a lack of the central neurotransmitter serotonin (68, 71, 72). Thus, people with chronic diseases have an increased risk of psychiatric morbidity.

Although our study is the most extensive population-based study examining the relationship between living alone and psychiatric morbidity to date, several limitations should be acknowledged. First, the design of this study was cross-sectional, and thus determining the duration of psychiatric morbidity in the people living alone is difficult. Further prospective studies are needed to elucidate the causal effects of living alone on psychopathology. Second, we used self-report questionnaires to assess psychiatric morbidity. As psychiatric disorders remain a social stigma, some people may have hesitated to answer truthfully, and thus we may have underestimated the prevalence of psychiatric morbidity. Furthermore, we may have underestimated the role of certain comorbid conditions due to a lack of information. Third, we defined psychiatric morbidity as a GAD-2 score ≧3, PHQ-2 score ≧3, or self-reported depression to include both depression and anxiety as the main focus of this study. However, we lacked data on self-reported anxiety. The initial questionnaire design of our study did not cover self-reported anxiety, so we used the GAD-2 score ≧3 to represent anxiety groups. Such an approach has been validated in other studies (73, 74). Fourth, we did not include some factors that may affect mental health, such as income, work, socioeconomic status, physical activity, and family support (75–77). This may have led to an underestimation of the risk of psychiatric morbidity and the association with living alone. Finally, only 16,582 participants completed the PHQ-2 and GAD-2, resulting in a lot of missing data. However, both PHQ-2 and GAD-2 are quantitative indicators which can represent the current status of participants, and by combining self-reported depression, PHQ-2 and GAD-2 can provide a holistic understanding of living alone and mental disorders.

## Conclusion

Our findings suggest that living alone is a risk factor for psychiatric morbidity, especially in those who are married. This highlights the importance of improving the care system for married persons living alone in concordance with divorce, separation or the death of a spouse to protect their physical and mental health. Further well-designed prospective studies are needed to investigate the causal effects of living alone on psychopathology.

## Data availability statement

The data analyzed in this study is subject to the following licenses/restrictions: the data underlying this study is from the Taiwan Biobank. Due to restrictions placed on the data by the Personal Information Protection Act of Taiwan, the minimal data set cannot be made publicly available. Data may be available upon request to interested researchers. Requests to access these datasets should be directed to S-CC, scarchenone@yahoo.com.tw.

## Ethics statement

The studies involving human participants were reviewed and approved by Institutional Review Board of Kaohsiung Medical University Hospital (KMUHIRB-E(I)-20210058). The patients/participants provided their written informed consent to participate in this study.

## Author contributions

Conceptualization, methodology, formal analysis, investigation, data curation, writing—review and editing, and visualization: J-HG and J-IL. Software and project administration: J-HG. Validation, supervision, and funding acquisition: S-CC. Resources: J-HG and S-CC. Writing—original draft preparation: T-YC. All authors have read and agreed to the published version of the manuscript.

## Funding

This work was supported partially by the Research Center for Environmental Medicine, Kaohsiung Medical University, Kaohsiung, Taiwan, from The Featured Areas Research Center Program within the framework of the Higher Education Sprout Project by the Ministry of Education (MOE) in Taiwan and by Kaohsiung Medical University Research Center Grant (KMU-TC111A01-1 and KMUTC111IFSP01).

## Conflict of interest

The authors declare that the research was conducted in the absence of any commercial or financial relationships that could be construed as a potential conflict of interest.

## Publisher's note

All claims expressed in this article are solely those of the authors and do not necessarily represent those of their affiliated organizations, or those of the publisher, the editors and the reviewers. Any product that may be evaluated in this article, or claim that may be made by its manufacturer, is not guaranteed or endorsed by the publisher.
