# Supplementary material for: Living alone is associated with a higher prevalence of psychiatric morbidity in a population-based cross-sectional study
Source: Front Public Health. 2022 Nov 17;10:1054615. doi: 10.3389/fpubh.2022.1054615 (PMC9714444; doi:10.3389/fpubh.2022.1054615)
Supplement: Supplementary file 1 [file Data_Sheet_1.docx]

**Supplementary Table 1. Parameter estimates of the association of psychiatric morbidity using least absolute shrinkage and selection operator (LASSO) regression**

| Parameter Estimates | | | | | | | | | | | | |
| --- | --- | --- | --- | --- | --- | --- | --- | --- | --- | --- | --- | --- |
| Parameter | DF | Estimate | Cross Validation Estimates | | | | | | | | | |
|  |  |  | 1 | 2 | 3 | 4 | 5 | 6 | 7 | 8 | 9 | 10 |
| Intercept | 1 | -0.063149 | 0.000107 | -0.000151 | -0.000242 | 0.0000768 | 0.000164 | 0.0000742 | -0.000164 | 0.00053 | -0.000399 | -0.000004 |
| Age | 1 | -0.000260 | -0.003914 | -0.004237 | -0.003760 | -0.0041009 | -0.004668 | -0.0040792 | -0.003876 | -0.00477 | -0.004230 | -0.004277 |
| Gender | 1 | 0.030794 | 0.016267 | 0.016306 | 0.015806 | 0.0163643 | 0.016191 | 0.0160530 | 0.016047 | 0.01585 | 0.015799 | 0.016047 |
| Body mass index | 1 | -0.000395 | -0.005836 | -0.005103 | -0.005252 | -0.0049902 | -0.005658 | -0.0051275 | -0.004759 | -0.00535 | -0.005517 | -0.005514 |
| Waist circumference | 1 | 0.000287 | 0.007606 | 0.006557 | 0.006756 | 0.0065624 | 0.007250 | 0.0063936 | 0.006588 | 0.00652 | 0.007298 | 0.006830 |
| Smoking | 1 | 0.021982 | 0.010689 | 0.010388 | 0.010026 | 0.0108696 | 0.010655 | 0.0106084 | 0.010850 | 0.01067 | 0.010298 | 0.010293 |
| Marital_status | 1 | -0.003307 | -0.001848 | -0.001195 | -0.001717 | -0.0013983 | -0.001486 | -0.0010180 | -0.001408 | -0.00148 | -0.001512 | -0.001461 |
| Educational_status | 1 | -0.003299 | -0.002700 | -0.002654 | -0.002453 | -0.0028794 | -0.003060 | -0.0024631 | -0.002576 | -0.00235 | -0.002664 | -0.002777 |
| Hypertension | 1 | 0.004701 | 0.002533 | 0.001828 | 0.002341 | 0.0021004 | 0.002099 | 0.0020282 | 0.001804 | 0.00236 | 0.002163 | 0.001999 |
| Diabetes | 1 | 0.010635 | 0.002604 | 0.002851 | 0.002798 | 0.0027943 | 0.002629 | 0.0026911 | 0.002370 | 0.00275 | 0.002363 | 0.002844 |
| Dyslipidemia | 1 | 0.015072 | 0.004032 | 0.004413 | 0.003996 | 0.0042836 | 0.004227 | 0.0043777 | 0.004339 | 0.00470 | 0.004079 | 0.004138 |
| CAD | 1 | 0.017025 | 0.002382 | 0.002323 | 0.002389 | 0.0024710 | 0.002387 | 0.0021707 | 0.002283 | 0.00238 | 0.002145 | 0.002367 |
| Valvular_heart_disea | 1 | 0.027660 | 0.005555 | 0.005154 | 0.005853 | 0.0059393 | 0.005861 | 0.0059988 | 0.006170 | 0.00657 | 0.005793 | 0.005754 |
| Asthma | 1 | 0.028007 | 0.005206 | 0.005414 | 0.005702 | 0.0051695 | 0.005911 | 0.0057361 | 0.005456 | 0.00585 | 0.005568 | 0.005377 |
| COPD | 1 | 0.023165 | 0.002693 | 0.003371 | 0.002584 | 0.0033655 | 0.002675 | 0.0031805 | 0.002848 | 0.00267 | 0.002543 | 0.002621 |
| Osteoporosis | 1 | 0.018936 | 0.004034 | 0.004195 | 0.003784 | 0.0042712 | 0.003906 | 0.0044141 | 0.003890 | 0.00448 | 0.004137 | 0.004483 |
| Gout | 1 | -0.003890 | -0.001520 | -0.001027 | -0.001264 | -0.0011689 | -0.001516 | -0.0013010 | -0.001349 | -0.00143 | -0.001166 | -0.001203 |
| GERD | 1 | 0.028493 | 0.010170 | 0.009507 | 0.010472 | 0.0098933 | 0.010348 | 0.0096059 | 0.010480 | 0.01021 | 0.009525 | 0.010123 |
| Peptic_ulcer | 1 | 0.024899 | 0.008794 | 0.009115 | 0.009074 | 0.0095137 | 0.009125 | 0.0093920 | 0.008830 | 0.00915 | 0.008999 | 0.009032 |
| IBS | 1 | 0.060088 | 0.009882 | 0.010156 | 0.009933 | 0.0096905 | 0.009266 | 0.0093728 | 0.009867 | 0.00971 | 0.010058 | 0.009517 |
| Parkinsons_Disease | 1 | 0.073174 | 0.003112 | 0.003219 | 0.002646 | 0.0022362 | 0.003011 | 0.0023967 | 0.002603 | 0.00275 | 0.003319 | 0.003253 |
| Dementia | 1 | 0.190803 | 0.004197 | 0.003735 | 0.003317 | 0.0043673 | 0.003674 | 0.0042679 | 0.003815 | 0.00388 | 0.003184 | 0.003085 |
| Schizophrenia | 1 | 0.169901 | 0.007581 | 0.008261 | 0.007915 | 0.0084321 | 0.007846 | 0.0077757 | 0.008469 | 0.00801 | 0.006635 | 0.007745 |
| Substance_abuse | 1 | 0.141925 | 0.003148 | 0.003088 | 0.003198 | 0.0034733 | 0.002824 | 0.0031391 | 0.003069 | 0.00372 | 0.002324 | 0.003243 |
| Living_alone | 1 | 0.025203 | 0.007581 | 0.007429 | 0.006537 | 0.0069681 | 0.007420 | 0.0075548 | 0.006848 | 0.00695 | 0.007268 | 0.007510 |

Abbreviations: CAD, coronary artery disease; COPD, chronic obstructive pulmonary disease; GERD, gastroesophageal reflux disease; IBS, irritable bowel syndrome; CKD, chronic kidney disease.

**Supplementary Table 2. Parameters associated with psychiatric morbidity in multivariate binary logistic analysis by gender**

| Parameters | **Male** |  | **Female** |  |
| --- | --- | --- | --- | --- |
|  | **Multivariable** |  | **Multivariable** |  |
|  | **OR (95% CI)** | ***p* value** | **OR (95% CI)** | ***p* value** |
| Age (per 1 year) | 0.998 (0.992 to 1.004) | 0.551 | 0.993 (0.989 to 0.997) | <0.001 |
| BMI (per 1 kg/m^2^) | 0.928 (0.897 to 0.959) | <0.001 | 0.975 (0.960 to 0.990) | 0.001 |
| WC (per 1 cm) | 1.031 (1.018 to 1.044) | <0.001 | 1.012 (1.006 to 1.018) | <0.001 |
| Smoking status, ever (*vs.* never) | 1.407 (1.249 to 1.586) | <0.001 | 2.045 (1.874 to 2.231) | <0.001 |
| Drinking status, ever (*vs.* never) | 0.965 (0.838 to 1.111) | 0.619 | 1.252 (1.068 to 1.468) | 0.006 |
| Regular exercise, yes (*vs.* no) | 0.922 (0.820 to 1.038) | 0.179 | 1.011 (0.942 to 1.084) | 0.766 |
| Marital status, married (*vs.* no) | 0.544 (0.461 to 0.642) | <0.001 | 1.088 (0.982 to 1.206) | 0.108 |
| Education status, ≧College (*vs.* no) | 0.993 (0.893 to 1.105) | 0.903 | 0.899 (0.849 to 0.952) | <0.001 |
| Hypertension, yes (*vs.* no) | 1.410 (1.190 to 1.671) | <0.001 | 1.196 (1.074 to 1.333) | 0.001 |
| Diabetes mellitus, yes (*vs.* no) | 1.219 (0.997 to 1.491) | 0.054 | 1.276 (1.107 to 1.471) | 0.001 |
| Dyslipidemia, yes (*vs.* no) | 1.410 (1.190 to 1.671) | <0.001 | 1.332 (1.185 to 1.497) | <0.001 |
| CAD, yes (*vs.* no) | 1.312 (0.978 to 1.759) | 0.070 | 1.536 (1.161 to 2.031) | 0.003 |
| Valvular heart disease, yes (*vs.* no) | 1.506 (1.152 to 1.968) | 0.003 | 1.568 (1.396 to 1.760) | <0.001 |
| Asthma, yes (*vs.* no) | 1.524 (1.207 to 1.925) | <0.001 | 1.633 (1.427 to 1.868) | <0.001 |
| COPD, yes (*vs.* no) | 1.358 (0.958 to 1.925) | 0.086 | 1.547 (1.220 to 1.963) | <0.001 |
| Osteoporosis, yes (*vs.* no) | 1.765 (1.328 to 2.346) | <0.001 | 1.359 (1.198 to 1.541) | <0.001 |
| Gout, yes (*vs.* no) | 0.926 (0.769 to 1.116) | 0.420 | 0.621 (0.402 to 0.960) | 0.032 |
| GERD, yes (*vs.* no) | 1.750 (1.526 to 2.006) | <0.001 | 1.625 (1.500 to 1.761) | <0.001 |
| Peptic ulcer, yes (*vs.* no) | 1.444 (1.263 to 1.651) | <0.001 | 1.688 (1.557 to 1.831) | <0.001 |
| IBS, yes (*vs.* no) | 2.699 (2.191 to 3.325) | <0.001 | 2.248 (1.945 to 2.598) | <0.001 |
| CKD, yes (*vs.* no) | 0.979 (0.707 to 1.355) | 0.898 | 1.161 (0.876 to 1.538) | 0.300 |
| Parkinson's Disease, yes (*vs.* no) | 2.171 (0.917 to 5.142) | 0.078 | 3.079 (1.555 to 6.100) | 0.001 |
| Dementia, yes (*vs.* no) | 8.173 (2.454 to 27.215) | 0.001 | 4.543 (1.612 to 12.803) | 0.004 |
| Schizophrenia, yes (*vs.* no) | 6.815 (4.239 to 10.957) | <0.001 | 4.331 (2.777 to 6.755) | <0.001 |
| Substance abuse, yes (*vs.* no) | 4.392 (1.841 to 10.479) | 0.001 | 3.851 (0.727 to 20.401) | 0.113 |
| **Living alone, yes (*vs.* no)** | 1.458 (1.212 to 1.753) | <0.001 | 1.620 (1.466 to 1.790) | <0.001 |

Values expressed as odds ratio (OR) and 95% confidence interval (CI). Abbreviations are the same as in supplementary Table 1.

Covariates in the multivariable model included age, body mass index, waist circumference, smoking status, drinking status, exercise habit, married status, educational status, hypertension, diabetes mellitus, dyslipidemia, coronary artery disease, valvular heart disease, asthma, chronic obstructive pulmonary disease, osteoporosis, gout, gastroesophageal reflux disease, peptic ulcer, irritable bowel syndrome, chronic kidney disease, Parkinson's Disease, dementia, schizophrenia and substance abuse.

**Supplementary Table 3. Odds ratios for psychiatric morbidity by marital status**

| Characteristics | No. of psychiatric morbidity cases / No. of subjects (%) | Adjusted odds ratio (95% CI) | *p value* |
| --- | --- | --- | --- |
| **All subjects, unmarried (n = 16,542)** | | | |
| Living alone (-) | 619/12,123 (5.1) | reference | - |
| Living alone (+) | 259/4419 (5.9) | 1.155 (0.990 to 1.348) | 0.067 |
| **All subjects, married (n = 105,059)** | | | |
| Living alone (-) | 4066/99,650 (4.1) | reference | - |
| Living alone (+) | 470/5409 (8.7) | 1.876 (1.692 to 2.081) | <0.001 |

CI = Confidence interval.

Covariates in the multivariable model included age, gender, body mass index, waist circumference, smoking status, educational status, hypertension, diabetes mellitus, dyslipidemia, coronary artery disease, valvular heart disease, asthma, chronic obstructive pulmonary disease, osteoporosis, gout, gastroesophageal reflux disease, peptic ulcer, irritable bowel syndrome, Parkinson's Disease, dementia, schizophrenia and substance abuse.

**Supplementary table 4. Odds ratios for psychiatric morbidity by marital status and gender**

| Characteristics | No. of psychiatric morbidity cases / No. of subjects (%) | Adjusted odds ratio (95% CI) | *p value* |
| --- | --- | --- | --- |
| **All subjects, unmarried (n = 16,542)** | | | |
| Living alone (-) | 619/12,123 (5.1) | reference | - |
| Living alone (+) | 259/4419 (5.9) | 1.156 (0.991 to 1.349) | 0.066 |
| **All subjects, married (n = 105,059)** | | | |
| Living alone (-) | 4066/99,650 (4.1) | reference | - |
| Living alone (+) | 470/5409 (8.7) | 1.879 (1.694 to 2.084) | <0.001 |
| **Male, unmarried (n = 5906)** | | | |
| Living alone (-) | 209/4348 (4.8) | reference | - |
| Living alone (+) | 91/1558 (5.8) | 1.234 (0.950 to 1.603) | 0.115 |
| **Male, married (n = 37,793)** | | | |
| Living alone (-) | 1006/36,351 (2.8) | reference | - |
| Living alone (+) | 69/1442 (4.8) | 1.684 (1.304 to 2.174) | <0.001 |
| **Female, unmarried (n = 10,636)** | | | |
| Living alone (-) | 410/7550 (5.4) | reference | - |
| Living alone (+) | 168/2861 (5.9) | 1.107 (0.913 to 1.343) | 0.300 |
| **Female, married (n = 67,266)** | | | |
| Living alone (-) | 3060/63,299 (4.8) | reference | - |
| Living alone (+) | 401/3967 (10.1) | 1.891 (1.687 to 2.120) | <0.001 |

CI = Confidence interval.

Covariates in the multivariable model included age, body mass index, waist circumference, smoking status, drinking status, exercise habit, married status, educational status, hypertension, diabetes mellitus, dyslipidemia, coronary artery disease, valvular heart disease, asthma, chronic obstructive pulmonary disease, osteoporosis, gout, gastroesophageal reflux disease, peptic ulcer, irritable bowel syndrome, chronic kidney disease, Parkinson's Disease, dementia, schizophrenia and substance abuse.

**Supplementary Table 5. Post-hoc analysis of the association between living alone and psychiatric morbidity**

| Characteristics | No. of psychiatric morbidity cases / No. of subjects (%) | Adjusted odds ratio (95% CI) | *p value* |
| --- | --- | --- | --- |
| Living alone (-) and married | 4066/99,650 (4.1) | reference | - |
| Living alone (-) and unmarried | 619/12,123 (5.1) | 1.269  (1.154 to 1.396) | <0.001 |
| Living alone (+) and unmarried | 259/4419 (5.9) | 1.437  (1.257 to 1.643) | <0.001 |
| Living alone (+) and married | 74/1088 (6.8) | 1.738  (1.364 to 2.213) | <0.001 |
| Living alone (+) and widowing | 120/1392 (8.6) | 1.760  (1.446 to 2.142) | <0.001 |
| Living alone (+) and divorce or separation | 276/2929 (9.4) | 2.058  (1.803 to 2.348) | <0.001 |

CI = Confidence interval.

Covariates in the multivariable model included age, gender, body mass index, waist circumference, smoking status, educational status, hypertension, diabetes mellitus, dyslipidemia, coronary artery disease, valvular heart disease, asthma, chronic obstructive pulmonary disease, osteoporosis, gout, gastroesophageal reflux disease, peptic ulcer, irritable bowel syndrome, Parkinson's Disease, dementia, schizophrenia and substance abuse.

| 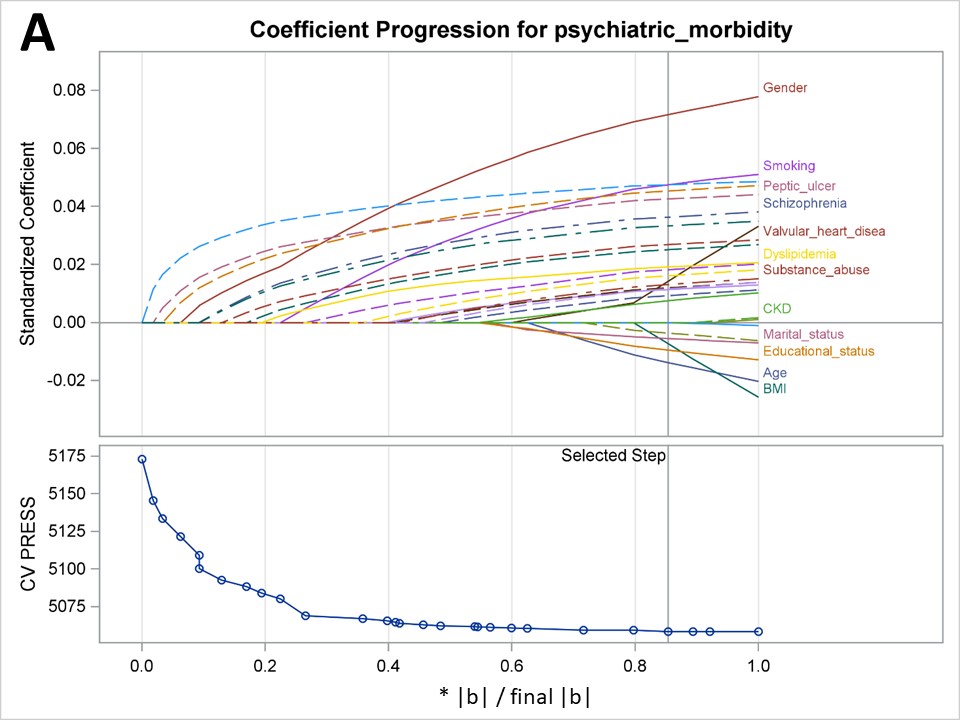 |
| --- |
| 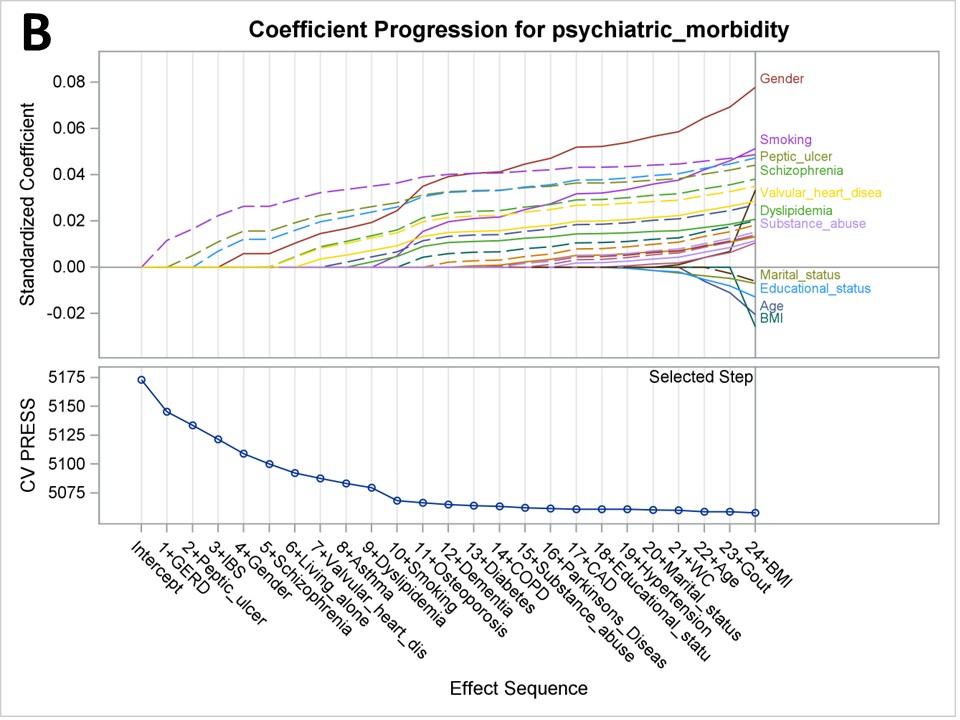 |

**Supplementary Figure 1. Results of the least absolute shrinkage and selection operator (LASSO) regression. A.** The figure shows the regression coefficient plot with 27 potential confounders by LASSO. **B.** The figure shows the effect sequence of the remaining 24 variables.

* The |b| means the beta value of the residual. When the beta value of the residual is closer to 0, it means that the model is more fit, and the more predictable the variable is to predict y (outcome). The first point is the intercept = 0, since there is no beta value. The second point is the variable which has the minimum |b| / Final |b|, and so on.

Abbreviation: CAD, coronary artery disease; COPD, chronic obstructive pulmonary disease; GERD, gastroesophageal reflux disease; IBS, irritable bowel syndrome; CKD, chronic kidney disease; WC, waist circumference; CV, predicted residual sum of square with k-fold cross validation; PRESS: predicted residual sum of squares.
